# Oxygen as a Virulence Determinant in Polymicrobial Infections

**DOI:** 10.1128/mBio.01249-16

**Published:** 2016-08-16

**Authors:** Elizabeth M. Selleck, Michael S. Gilmore

**Affiliations:** a Department of Ophthalmology, Harvard Medical School, Massachusetts Eye and Ear Infirmary, Boston, Massachusetts, USA; b Department of Microbiology and Immunobiology, Harvard Medical School, Boston, Massachusetts, USA

## Abstract

Infections caused by multiple organisms, or polymicrobial infections, are likely more common than is broadly appreciated. Interaction among microbial communities (and with their host) can change the infection landscape by subverting immunity, providing nutrients and inhibiting competing microbes. Stacy et al. (A. Stacy, D. Fleming, R. J. Lamont, K. P. Rumbaugh, and M. Whiteley, mBio 7:e00782-16, 2016, http://dx.doi.org/10.1128/mBio.00782-16) described a novel mechanism that results in synergistic growth of oral microbes *Aggregatibacter actinomycetemcomitans* and *Streptococcus gordonii*. The authors used whole-genome fitness profiling by transposon sequencing (Tn-seq) to identify genes differentially required for growth *in vitro* versus in a mono- or coinfection in a thigh abscess model. They found that coinfection with *S. gordonii* allowed *A. actinomycetemcomitans* to shift from an anaerobic to an aerobic mode of growth. This shift involved the production of a terminal electron acceptor H_2_O_2_ by *S. gordonii* and increased *A. actinomycetemcomitans* persistence—an interaction termed “cross-respiration.”

## COMMENTARY

**FIG 1:**
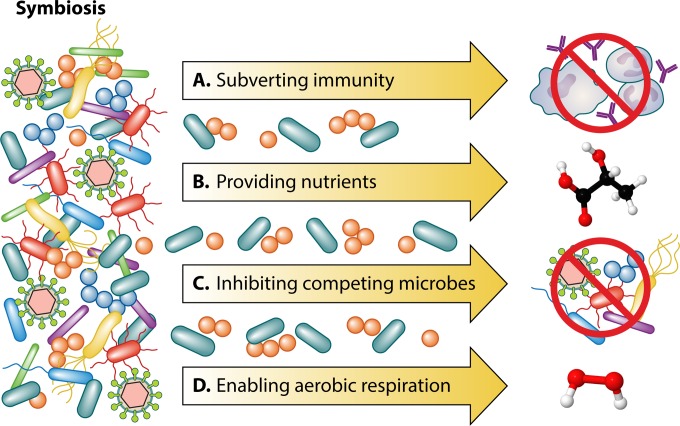
In a polymicrobic infection, one microbe can promote the virulence of a coinfecting microbe in several ways, including (A) subverting host defenses by limiting killing by phagocytosis or complement activity; (B) providing novel carbon sources or nutrients at the site of infection; (C) inhibiting the growth of competing microbes, allowing pathogen overgrowth; or (D) providing novel terminal electron acceptors, allowing more-efficient energy production.

It has long been known that one microbe—virus, fungus or bacterium—can change the landscape of a host tissue or infection environment that enhances the ability of another microbe to cause disease. The most famous example is the tremendous mortality caused by *Streptococcus pneumoniae* in patients infected by the 1918 H1N1 influenza virus (1). However, in few cases is the precise nature of the polymicrobic interaction that exacerbates infection known. Altermeier described an early example of polymicrobial synergy in 1941. He showed increased virulence of several anaerobic bacterial species (including *Bacteroides fragilis*) coinfected with aerobic bacterial species (such as *Escherichia coli*) isolated from the same peritoneal abscess in a subcutaneous abscess guinea pig model compared to infections caused by the anaerobe alone (2). Suggested mechanisms of enhanced virulence of coinfecting organisms include prevention of phagocytosis by host immune cells, reduction of oxygen-reduction potentials in the host tissue, and the production of a nutrient or metabolite by one organism that promotes the growth of the other organism (reviewed in reference 3) (Fig. 1).

*Aggregatibacter actinomycetemcomitans* and *Streptococcus gordonii* are found in plaque coaggregates in the oral cavity (4) and are capable of causing abscesses in the gingiva, skin, brain, and lung (5). In earlier work, the authors found that production of lactate and H_2_O_2_ by *S. gordonii* contributed to synergy between these organisms *in vitro* and in a murine thigh abscess model of infection (6). Lactate is a preferred carbon source for *A. actinomycetemcomitans* and a metabolic by-product of *S. gordonii* which allows complementary utilization of available glucose in the abscess. Additionally, the production of H_2_O_2_ by *S. gordonii* induces expression of a complement resistance factor in *A. actinomycetemcomitans*, reducing its susceptibility to this host defense factor (7). Both lactate use by *A. actinomycetemcomitans* and H_2_O_2_ production by *S. gordonii* require molecular oxygen; however, previous transcriptional profiling of *A. actinomycetemcomitans* in monoinfected abscess suggested that *A. actinomycetemcomitans* metabolism is largely anaerobic in this setting (8).

Stacy et al. investigated this paradox in their recent work (9). The authors used transposon sequencing (Tn-seq), in which transposon insertion mutants were used to identify essential genes in *A. actinomycetemcomitans* in different environments. To define the metabolic state of *A. actinomycetemcomitans* in a coinfected abscess, the authors compared the essential genes identified in *A. actinomycetemcomitans* under several sets of conditions, including during oxic and anoxic growth *in vitro*, as well as for growth and survival in monoinfection and in coinfection with *S. gordonii* in an abscess. A distinct set of essential genes were found under anoxic and oxic growth conditions. These fitness determinants were then compared to those required for *A. actinomycetemcomitans* growth and survival in an abscess during a monoinfection. While both oxic and anoxic fitness determinates were identified as essential in a monoinfection, more genes overlapped under conditions of anoxic *in vitro* growth, suggesting that *A. actinomycetemcomitans* metabolism is largely anaerobic in a monoinfected abscess. The final set of comparisons consisted of comparisons of *A. actinomycetemcomitans* in a coinfection with *S. gordonii* in the abscess infection model to monoinfection conditions and *in vitro* conditions. Coinfection revealed a dramatic shift in fitness determinants in *A. actinomycetemcomitans* compared to monoinfection; fewer traits identified as virulence factors were required, and fewer functions involved in the acquisition of carbon and biosynthesis were needed. Moreover, genes required for aerobic growth, including genes encoding aerobic formate dehydrogenase and components of the quinone biosynthesis pathway, were also required during coinfection, implying a switch to an aerobic respiration-like state by *A. actinomycetemcomitans*. This implication was supported by the increase in the number of fitness determinants shared between oxic growth *in vitro* and coinfection compared to monoinfection. The switch to an aerobic respiration-like state was underscored by utilization of H_2_O_2_ as a terminal electron acceptor by *A. actinomycetemcomitans* and its ability to generate a suitable proton gradient in the absence of ATP biosynthesis in mixed infection but a reduced ability to do this as the sole pathogen.

The utilization of a terminal electron acceptor generated by a coinfecting microbe, termed cross-respiration, has many interesting implications for understanding observation of polymicrobial synergy in other infections. Work by Winter et al. (10) recently described a similar case involving *Salmonella enterica* serovar Typhimurium in the inflamed gut. They found that the generation of tetrathionate, which was formed by oxidation of sulfur compounds produced by commensal microbes by reactive oxygen species (ROS) generated by the host, enhanced *S*. Typhimurium growth. As *S*. Typhimurium is unusual among microbes in the intestinal lumen in its ability to use it as a terminal electron acceptor, the formation of tetrathionate promoted growth and subsequent invasion by *S*. Typhimurium of the gut epithelium (10). Similar facilitation of *S*. Typhimurium infection in the presence of oxygen was also seen after depletion of butyrate-producing clostridia following antibiotic treatment. Decreases in butyrate levels following loss of clostridia promoted the utilization of glucose by colonocytes, increasing local oxygen levels and allowing *S*. Typhimurium aerobic respiration and proliferation and in turn leading to increased fecal-oral transmission in mice (11). *S*. Typhimurium cytochrome *bd*-II oxidase mutants do not benefit from the presence of oxygen as a terminal electron acceptor and were far less likely to be spread by fecal-oral transmission. As predicted by this mechanism, oxygen-driven proliferation of *S*. Typhimurium can be prevented by replacing butyrate with tributyrin as an alternate source of energy for colonocytes (11).

Production of ROS such as H_2_O_2_ affects the transcriptional programs of other microbes residing in the otherwise-reduced oxygen environment of the gut, such as the murine gut pathogen *Citrobacter rodentium*. In mice lacking NADPH oxidase that cannot produce ROS, microbes, including protective lactobacilli, proliferate. Lactobacilli, like *S. gordonii*, are endogenous producers of H_2_O_2_ and contribute a protective barrier in the gut. In the absence of oxygen species, *C. rodentium* produce a number of virulence factors leading to disease. However, production of H_2_O_2_ by lactobacilli reduces *C. rodentium* expression of virulence factors encoded in the locus of enterocyte effacement (LEE) (12). This infection model demonstrates virulence inhibition by the availability of a terminal electron acceptor but nevertheless illustrates that oxygen species production by one member of a consortium can alter the virulence of another, modifying gut homeostasis.

The use of Tn-seq to gain insights into the *in situ* production of a terminal electron acceptor by another organism at the site of infection illustrates the value of genome-wide fitness profiling for understanding the metabolic state of an organism within an infection site. While Stacy et al. determined the respiratory state of *A. actinomycetemcomitans* using this technique, it has also been used to gain insights into the physiological state and nutritional requirements of *Staphylococcus aureus* in an abscess (13), the genes required for carriage and infection of *Streptococcus pneumoniae* (14), and the colonization factors required for intestinal infection by *Vibrio cholerae* (15), providing new insights of potential value for optimizing infectious disease treatment and therapeutic design. Stacy et al. demonstrated that synergistic growth in a mixed infection exacerbates pathogenesis and depends on the exchange of lactate and a terminal electron acceptor. These results imply that antimicrobial regimens that affect this aspect of *S. gordonii* physiology might aid in fighting these coinfections.
